# Gold Nanoparticle–Mediated Targeted Delivery of Recombinant Human Endostatin Normalizes Tumour Vasculature and Improves Cancer Therapy

**DOI:** 10.1038/srep30619

**Published:** 2016-07-29

**Authors:** Wei Li, Xiaoxu Zhao, Bin Du, Xin Li, Shuhao Liu, Xiao-Yan Yang, Hui Ding, Wende Yang, Fan Pan, Xiaobo Wu, Li Qin, Yunlong Pan

**Affiliations:** 1 Department of General Surgery, the First Affiliated Hospital of Jinan University, Guangzhou 510632, China; 2 Department of Pathology, the First Affiliated Hospital of Jinan University, Guangzhou, 510632, China; 3 Department of Histology and Embryology, Medical School of Jinan University, Guangzhou 510632, China

## Abstract

Tumour vasculature is generally disordered because of the production of excessive angiogenic factors by tumour cells, which results in tumour progression and reduces the effectiveness of radiotherapy or chemotherapy. Transient anti-angiogenic therapies that regulate tumour vascular morphology and function and improve the efficiency of antitumour therapy are under investigation. Recombinant human endostatin (Endostar/rhES) is a vascular angiogenesis–disrupting agent that has been used to treat non-small cell lung cancer (NSCLC) in the clinical setting. In this study, we used gold nanoparticles (AuNPs) as a drug-delivery system (DDS) for targeted tumour delivery of rhES for short therapy, which resulted in transient tumour vascular normalization, reduced permeability and hypoxia, strengthened blood vessel integrity, and increased blood-flow perfusion. Moreover, combination therapy with 5-FU over this timeframe was substantially more effective than 5-FU monotherapy. In conclusion, our research demonstrates the potential use of AuNPs as a drug-delivery platform for transporting rhES into a tumour to induce transient tumour vascular normalization and enhance the antitumour efficacy of cytotoxic drugs.

Solid tumour growth and progression requires sufficient oxygen and nutrients and thus depends on the sprouting of new tumour vessels from existing vessels^1^. This process, known as angiogenesis, is initiated by a wealth of pro-angiogenic factors produced by tumour cells in response to a hypoxic microenvironment^2^. Excessive, continuous production of pro-angiogenic factors leads to an imbalance between pro- and anti-angiogenic signals^3^, which results in structurally and functionally abnormal vessels characterized by dilatation, tortuosity, high permeability, and risk of recurrence or metastasis. Based on theoretical models and preclinical studies, anti-angiogenesis therapies that “starve” tumour cells and induce tumour cell death have been proposed. A series of tumour angiogenesis inhibitors [such as Bevacizumab (Avastin^®^), Sorafenib (Nexavar^®^), and Sunitinib (Sutent^®^)] have been approved for clinical application by the Food and Drug Administration (FDA)^4^, and nearly all of these agents are directed against the VEGF-A/VEGFR2 signalling pathway.

However, some researchers have observed that anti-angiogenic agents may enhance tumour invasiveness and metastasis in preclinical models^5^, and results from clinical trials with anti-angiogenic monotherapy have been disappointing, with failure to provoke significant response rates or improve overall survival^6^. In response, the new concept of “tumour vascular normalization” has been proposed by Jain and colleagues^7^. This theory suggests that anti-angiogenesis therapy may reduce blood flow to the tumour and increase intratumour hypoxia, thereby resulting in chemo/radioresistance. However, further studies have demonstrated that the application of moderate doses of anti-angiogenesis agents can normalize abnormal tumour vascularization for a short period and improve blood perfusion and tumour therapy^8^,^9^,^10^.

Endostatin, a 20-kDa C-terminal non-collagenous fragment of type XVIII collagen, inhibits angiogenesis in different pathological conditions^11^. Moreover, endostatin specifically binds to nucleolin on the cell surface to inhibit tumour lymphangiogenesis and lymphatic metastasis^12^ by down-regulating VEGF-C levels and VEGFR-3 gene expression^13^. Recombinant human endostatin (Endostar or rhES) is a modified human endostatin in which a nine-amino acid sequence (MGGSHHHHH) added to the N-terminus of endostatin confers a more stable structure while retaining endogenous endostatin function^14^.

To improve *in vivo* efficiency and reduce dosing frequency, a variety of drug-delivery systems (DDSs) have been developed. Gold nanoparticles (AuNPs), as a nanoparticle DDS, exhibit unique properties, including chemical stability, inherent biocompatibility, low cytotoxicity^15^, and the capability of delivering various biological molecules to their targets, including small drug molecules, peptides, proteins, and nucleic acids such as DNA or RNA^16^,^17^. AuNPs can become trapped in solid tumours and be retained at high concentrations for prolonged periods^18^ due to the enhanced permeation and retention (EPR) effect, which is likely associated with the heterogeneity of tumour blood vessels^19^. The width between the endothelial cells in tumour vessels is ranging from 200 nm to 1.2 μm^20^; therefore, nanoscale drugs can extravasate and diffuse from tumour vessels but not normal vessels due to the leaky nature of tumour vessels^20^,^21^. Furthermore, vessels with excessive leakiness increase interstitial fluid pressure inside tumours and cause interstitial fluid to ooze from the tumour into the surrounding tissues, resulting in decreased drug concentrations and the spread of tumour cells^22^.

In this study, we used AuNPs as a drug carrier to deliver rhES into a tumour via passive targeting and facilitate transient vascular normalization to improve antitumour therapeutic efficacy.

## Results

### Characterization of rhES-AuNPs-PEG

**Figure 1:**
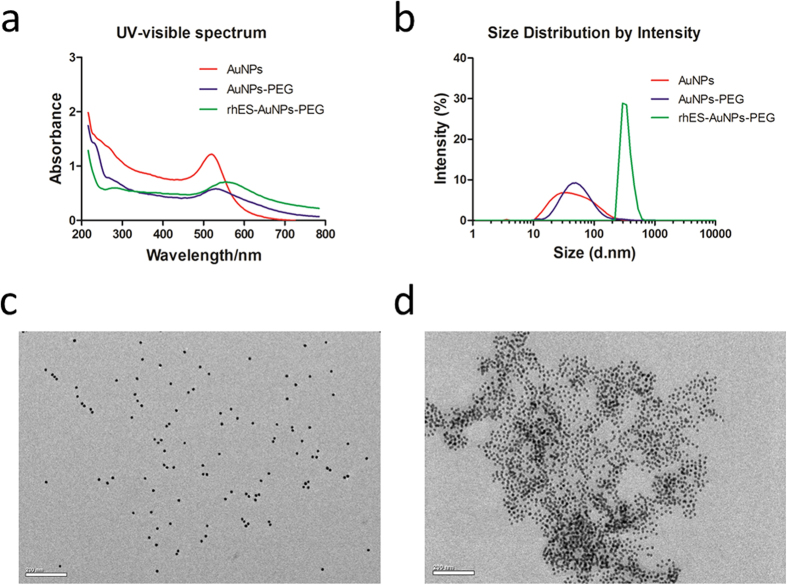
Characterization of rhES-AuNPs-PEG. (**a**) UV-visible spectra of bare AuNPs, PEG-, and rhES-PEG-functionalized AuNPs. The UV-visible spectra of AuNPs and rhES-AuNPs-PEG were characterized by SPR absorption peaks at approximately 517 nm and 550 nm, respectively. (**b**) The hydrodynamic size distributions of AuNPs, PEG-, and rhES-PEG-functionalized AuNPs were 58.2 ± 7.1 nm, 70.6 ± 6.8 and 337.9 ± 13.0 nm, respectively. (**c**,**d**) TEM imaging revealed discrete particles in AuNPs and slightly aggregated particles in rhES-AuNPs-PEG. Scale bars: 200 nm.

The UV-visible spectrum of AuNPs revealed a characteristic surface plasmon resonance (SPR) absorption band at approximately 517 nm; after combination with rhES, the compound exhibited a peak at 550 nm, which was attributed to the introduction of groups on the surface of the AuNPs (Fig. 1a). Dynamic light scattering (DLS) indicated that the hydrodynamic diameters of AuNPs, PEG-, and rhES-PEG-functionalized AuNPs were 58.2 ± 7.1 nm, 70.6 ± 6.8 and 337.9 ± 13.0 nm, respectively (Fig. 1b), and the zeta potentials were −39.3 ± 1.1 mV, −16.5 ± 0.5 mV and −13.7 ± 1.0 mV, respectively. Field emission transmission electron microscopy (FETEM) images revealed that the AuNPs were well-dispersed spherical particles that were uniform in size (Fig. 1c). The rhES-AuNPs-PEG conjugates exhibited a slightly aggregated structure (Fig. 1d), consistent with the bathochromic shift in the UV-visible spectrum.

### Loading efficacy and release rate

**Figure 2:**
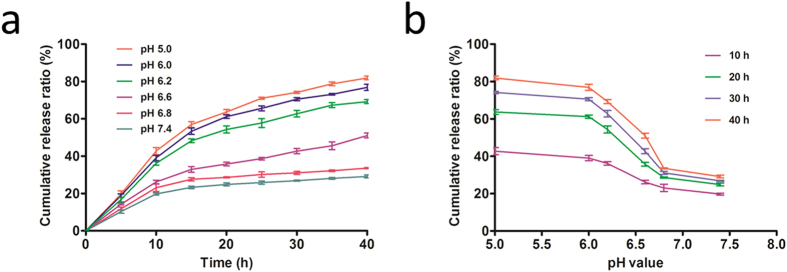
*In vitro* release of rhES from AuNPs. (**a**) The rhES release profile was two-phase, with an initial quick release followed by continuous release. Release was higher and more rapid at low pH (pH 5.0 and 6.0) than at pH 6.8 and 7.4, indicating that the rhES release ratio from AuNPs was pH dependent. (**b**) The linear relationship between pH and release ratio. Different time points and pH values altered the release ratio; overall, low pH values promoted the release of the drug.

The free rhES in the supernatant was measured as described previously, and the loading of rhES on AuNPs was determined to be 86%. The release of rhES at pH 6.8 and 7.4 was slow and sustained; however, the release ratio in acidic media (pH 5.0 and 6.0) continuously increased with time, and the release capability was increased gradually with the reduction of the pH value (pH 6.6 and 6.2). Thus, low pH resulted in more rapid release of rhES compared with release at pH 6.8 (*P* = 0.0091) and 7.4 (*P* = 0.0040) (Fig. 2a). The linear relationship between pH and the release ratio is presented in Fig. 2b; over different time periods, the ratio of rhES released from AuNPs was strongly dependent on pH. This pH-dependent release might enhance the selectivity of tumour targeting because the acidic conditions of the tumour microenvironment may trigger the release of rhES from the AuNPs. Furthermore, the lower release rate at pH 7.4 would protect the stability of the carrier delivery system in the circulation and normal tissues, as observed in previous research^23^.

### Accumulation of rhES-AuNPs-PEG in tumours

**Figure 3:**
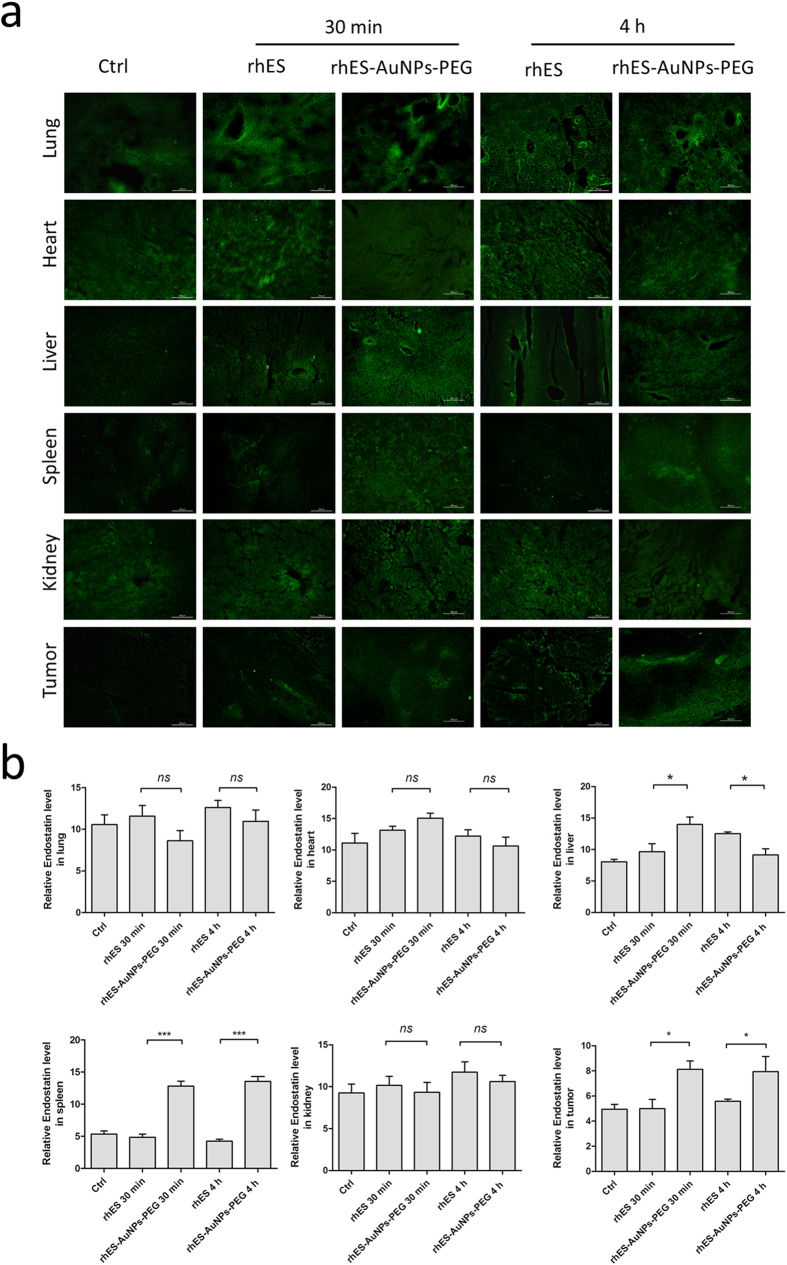
*In vivo* distribution of rhES in different organs at 30 min and 4 h. (**a**) rhES accumulation in tumours was much greater following treatment with rhES-AuNPs-PEG than with rhES (*P < 0.05) within 30 min after intravenous injection. (**b**) After 4 h, the rhES levels in tumours treated with rhES-AuNPs-PEG were maintained at high levels; however, the accumulation of rhES in tumours treated with rhES monotherapy still much lower than those treated with rhES-AuNPs-PEG (*P < 0.05).

After intravenous injection, rhES-AuNPs-PEG accumulated rapidly and specifically in tumours at a higher level than rhES alone at 30 min (*P* = 0.0141) and 4 h (*P* = 0.0332) (Fig. 3a,b). The difference in localization demonstrates that rhES-AuNPs-PEG exhibited improved tumour-targeting efficiencies and longer retention. However, rhES localization to the lung, heart, and kidney was not significantly different. A significant increase in rhES was observed in the liver (within 30 min) and spleen, which may be due to phagocytosis by the reticuloendothelial system (RES).

In addition, optical *in vivo* imaging of Cy5-labeled regents revealed that after the i.v. injection, rhES-AuNPs-PEG rapidly accumulated in tumours as early as 30 min and increased in tumours in 4 h (Fig. S1). By contrast, rhES accumulated to lower levels in tumours than rhES-AuNPs-PEG, and we observed poor specific localization *in vivo*. This finding confirms that AuNPs are excellent nanoparticle drug carriers that increase tumour targeting.

### Short-term rhES-AuNPs-PEG treatment facilitates transient vascular normalization

**Figure 4:**
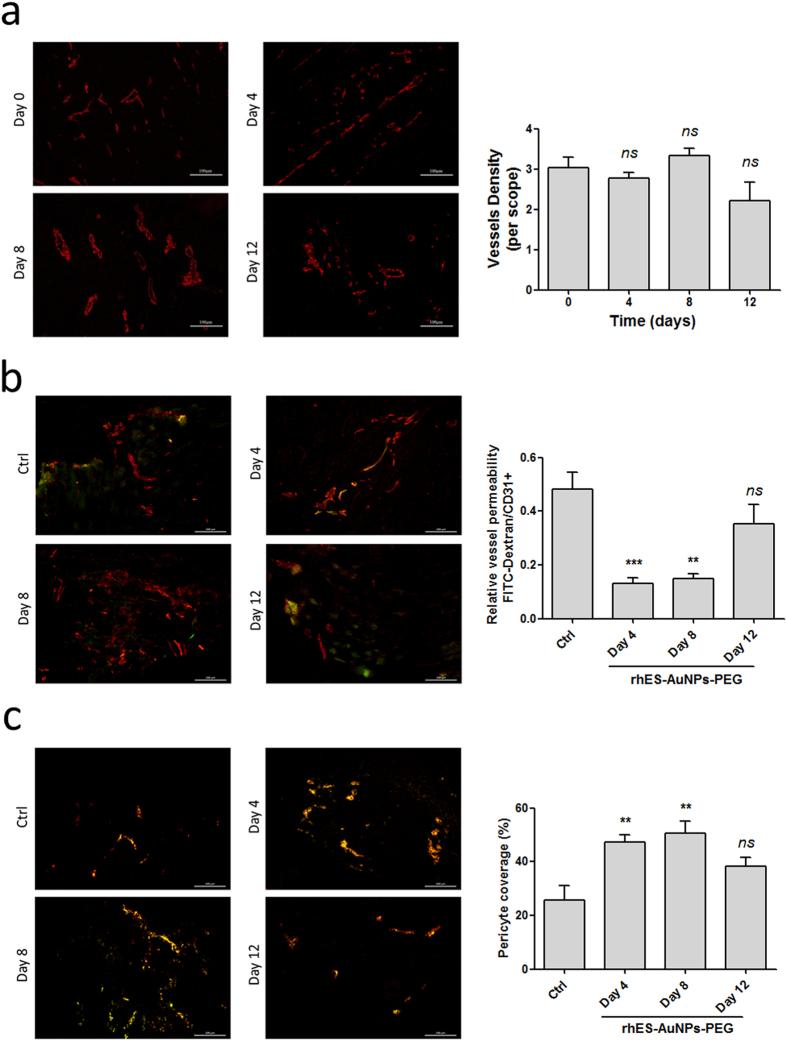
rhES-AuNPs-PEG improves vascular integrity and reduces leakage. (**a**) CD31 staining of tumour sections. Scale bars: 100 μm. (**b**) FITC-dextran and CD31 staining of leakage in tumours. The ratio of the FITC-stained area to CD31^+^ area was determined and is presented as the relative value (***P < 0.001; **P < 0.01). Scale bars: 200 μm. (**c**) Endothelial cells and pericytes were visualized by CD31 (red) and α-SMA (green) staining of tumours (**P < 0.01). Scale bars: 200 μm.

We next evaluated vascular normalization by measuring CD31 as a marker of the endothelium, α-smooth muscle actin (α-SMA) as a marker of pericytes, and FITC-labelled dextran as a marker of permeability. CD31 staining revealed that the vessels in the control treatment were chaotic and deformed (Fig. 4a). By contrast, after treatment with rhES-AuNPs-PEG for 4 days or 8 days, the tumour vessels appeared organized. The tumour vessels exhibited increased vessel diameter and improved structure, but similar effects were not observed after 12 days. Moreover, a significant difference in vascular density was not observed between day 0 and day 12. Our data indicate that rhES-AuNPs-PEG improved vascular morphology in tumours in a highly time-dependent manner.

The control tumours exhibited increased vascular permeability, which is associated with excessive deposition of dextran around the tumour vessels, as well as irregular CD31 staining. However, after treatment with rhES-AuNPs-PEG for 4 days and 8 days, leakage of dextran from vessels decreased (day 4, *P* = 0.0009; day 8, *P* = 0.0011; day 12, *P* = 0.2206; Fig. 4b). Additionally, double staining for CD31 and α-SMA demonstrated that the treatment increased the proportion of pericyte-covered vessels (day 4, *P* = 0.0078; day 8, *P* = 0.0082; day 12, *P* = 0.0815; Fig. 4c). These results indicate that rhES-AuNPs-PEG increased the integrity of the endothelium by increasing pericyte coverage, indicating that the tumour vessels were more mature.

### rhES-AuNPs-PEG increase blood perfusion and reduce hypoxia in tumour sections

**Figure 5:**
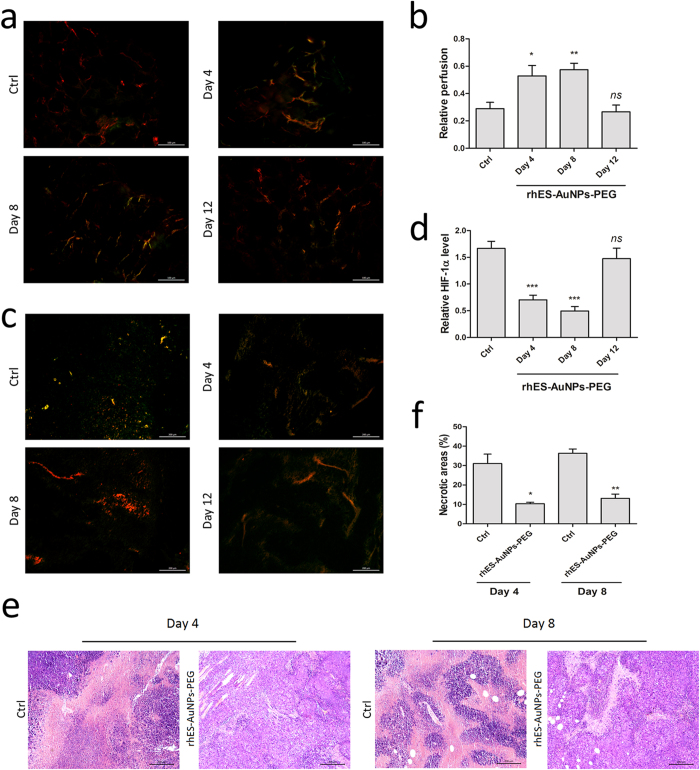
rhES-AuNPs-PEG increase blood perfusion and reduce hypoxia in tumour sections. (**a**,**b**) Lectin perfusion and CD31 staining of tumour sections. The lectin^+^ area is presented as the percentage of the CD31^+^ area (*P < 0.05; **P < 0.01). Scale bars: 200 μm. (**c**,**d**) The ratio of the HIF-1α area/total area of the tumour section was determined and is presented as the relative value (***P < 0.001). Scale bars: 200 μm. (**e**,**f**) Necrotic areas in tumours after treatment for different times versus controls (*P < 0.05; **P < 0.01) (haematoxylin and eosin staining). Scale bars: 200 μm.

Because rhES-AuNPs-PEG normalized the morphology of blood vessels, we next evaluated whether treatment could improve the function of the vessels. Vessel perfusion improved in tumours after 4 days of treatment, and perfusion gradually increased until day 8. However, an obvious decrease in perfusion was observed on day 12 (day 4, *P* = 0.0271; day 8, *P* = 0.0028; day 12, *P* = 0.7487; Fig. 5a,b). By contrast, vessels in the control tumours exhibited less perfusion during the entire course of treatment.

Because hypoxia triggers angiogenesis, we analysed tumour oxygenation by staining for the hypoxia marker hypoxia inducible factor-1α (HIF-1α), which enables tumour cells to produce a variety of pro-angiogenic factors^24^. Treatment with rhES-AuNPs-PEG relieved hypoxia in tumours (day 4, *P* = 0.0003; day 8, *P* < 0.0001; day 12, *P* = 0.4408; Fig. 5c,d), consistent with the increase in blood flow during the normalization window of the tumour vessels. Histological analysis revealed that the tumours in mice treated with rhES-AuNPs-PEG exhibited smaller necrotic areas in the tumoural regions (day 4, *P* = 0.0138; day 8, *P* = 0.0018; Fig. 5e,f). Together, these data indicate that treatment with rhES-AuNPs-PEG temporarily increases blood perfusion and alleviates hypoxia and necrosis in tumours.

### Pre-treatment with rhES-AuNPs-PEG increases intratumour drug delivery

**Figure 6:**
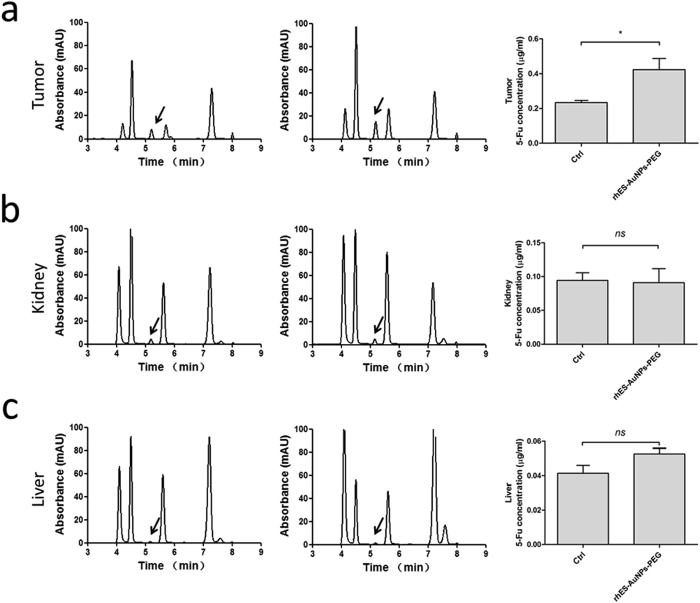
HPLC analysis of 5-FU in different organs. After intraperitoneal 5-FU injection, the concentration of 5-FU in tumours increased significantly in mice pre-treated with rhES-AuNPs-PEG compared with treatment with 5-FU alone (**a**) (*P < 0.05). rhES-AuNPs-PEG treatment did not increase 5-FU concentrations in the kidneys (**b**) or livers (**c**) compared with 5-FU alone.

To determine if vessel normalization increases chemotherapeutic efficacy, we administered 5-FU within and outside this normalization window. Compared with monotherapy, combination with rhES-AuNPs-PEG significantly increased 5-FU localization to the tumour sections (*P* = 0.0428; Fig. 6a) but had no effect on the kidneys (*P* = 0.8948; Fig. 6b) or liver (*P* = 0.1166; Fig. 6c), indicating that the normalization was tumour-specific.

### Treatment with rhES-AuNPs-PEG improves cytotoxic therapeutic outcomes

**Figure 7:**
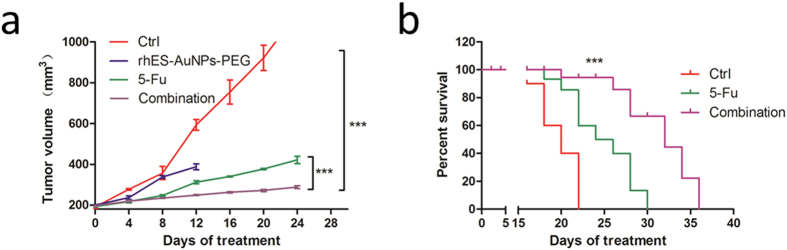
rhES-AuNPs-PEG enhances the antitumour efficacy of 5-FU. (**a**) Quantification of tumour volume at different times following implantation (***P < 0.001). (**b**) Comparison of overall survival. The *P* values were calculated using the log-rank test (***P < 0.001 vs. ctrl).

Treatment with 5-FU monotherapy and 5-FU combined with rhES-AuNPs-PEG reduced growth significantly (Fig. 7a). However, tumours receiving combination therapy exhibited a more significant reduction in growth on day 12 compared to tumours receiving 5-FU monotherapy (*P* = 0.0012), and this advantage continued until day 24 (*P* = 0.0025). This result is consistent with the results of the HPLC analysis, which revealed high concentrations of 5-FU in tumours after pre-treatment with rhES-AuNPs-PEG. The median survival time of mice treated with rhES-AuNPs-PEG plus 5-FU was 1.60-fold longer than that of the placebo group (Fig. 7b). These data suggest that tumour vascular normalization induced by short-term rhES-AuNPs-PEG treatment enhanced the efficacy of 5-FU.

## Discussion

Solid tumours are always associated with pathological angiogenesis, which is initiated by pro-angiogenic factors that are produced by tumour cells under hypoxic conditions. These newly formed vessels are structurally abnormal and exhibit dysfunctions that impair perfusion and oxygenation. Moreover, the changes in the tumour microenvironment promote tumour invasion and metastasis^25^, and hypoperfusion limits the delivery of therapeutic agents into the tumour, which results in insensitivity to chemo/radiotherapy^26^. Most studies have only concentrated on anti-angiogenesis to “starve” tumours using high-dose angiogenesis inhibitors; however, emerging evidence has revealed a contradiction between anti-angiogenesis and traditional chemotherapy. Lower doses of anti-angiogenesis reagents can induce tumour vascular normalization, which results in temporarily increased blood flow and improves the effectiveness of subsequent chemo/radiotherapy. In fact, a variety of evidence supports the existence of a normalization window.

Endostatin is a broad-spectrum endogenous angiogenesis inhibitor^27^,^28^ that suppresses angiogenesis via many pathways, such as bFGF/FGF-2, α5β1, E-selectin, metalloproteinases, and VEGF^27^. Furthermore, the recombinant form (Endostar/rhES) has been used in the clinical setting for the treatment of lung cancer^29^. In this study, we used AuNPs as a tool to specifically target tumour vessels for short therapy to induce tumour vascular normalization. AuNPs are excellent nano-drug carriers because of their large surface-area-to-volume ratio and biocompatibility and have been used successfully to deliver large biomacromolecules without compromising their activity^30^. Moreover, the grafting of methoxy polyethylene glycol-thiol (mPEG-Thiol) onto AuNPs after being combined with rhES to maintain colloidal stability^31^ and prevent nonspecific serum protein binding to the surface^32^,^33^ reduces clearance by macrophages of the RES^33^, consequently prolonging their circulation time *in vivo* and improving tumour uptake. Our data indicated that rhES was successfully directly bound to AuNPs (presumably via a noncovalent linkage), thus targeting delivery into tumours more efficiently. As previously mentioned, extravasation of nanoparticles, as a passive targeting method, is dependent on heterogeneous vessels^18^. The integrity of the vascular endothelium prevents macromolecules and nanoparticles from permeating into most normal tissues, and this selective extravasation effect favours a long circulation time and less toxicity.

Pericytes have been implicated as mediators of tumour angiogenesis and metastasis^34^. Pericytes normally envelope endothelial cells and coordinate intercellular signalling to establish direct cell–cell contact and support endothelial integrity, stabilization, and maturation^35^,^36^. The dissociation of pericytes and endothelial cells is the initial stage of tumour angiogenesis and facilitates endothelial cell migration and endothelial tubulogenesis^34^,^35^. Meanwhile, the detachment-mediated increase in vessel permeability leads to the leakage of plasma proteins^34^ and cancer cell metastasis into the circulation^36^. Several molecules are involved in pericyte-mediated interaction signalling, including PDGFRβs, VEGFRs, and Tie-2^36^,^37^,^38^. Moreover, disruption of pericytes and endothelial cells activates pericytes to secrete more VEGF and bFGF, establishing a vicious cycle of continuous angiogenesis while promoting endothelial cell proliferation and migration into the surrounding tissue^34^. In this study, our observations suggested that treatment with rhES-AuNPs-PEG improved pericyte coverage, indicating that the tumour blood vessels were more mature.

Hypoxia is an indicative feature of solid tumours and a potent inducer of tumour angiogenesis^39^,^40^. HIF-1α is the primary mediator of the hypoxia response and induces overexpression of pro-angiogenic factors produced by tumour and host cells under hypoxic conditions^39^; for example, VEGF is primarily regulated by HIF-1α at the transcriptional level^41^ and causes tumour vascular angiogenesis. By contrast, increasing vascular perfusion is associated with reduced tumour hypoxia, and our data corroborate these findings. After treatment with rhES-AuNPs-PEG, HIF-1α expression was significantly reduced in the tumours. Our result is consistent with published studies demonstrating that endostatin down-regulates the expression of HIF-1α in the microvascular endothelium^42^,^43^. However, some data suggest that the anti-angiogenic activity of endostatin is independent of the HIF-1/VEGF pathway *in vitro*^44^.

Next, we examined the concentration of 5-FU in tumours by HPLC analysis. Treatment with rhES-AuNPs-PEG increased the concentration of intratumour 5-FU, which is closely related to increased blood flow in tumours, and this improvement was obvious after 4 and 8 days of treatment with rhES-AuNPs-PEG. Moreover, 5-FU combined with tumour vascular normalization therapy reduced disease progression.

In summary, we prepared a tumour-specific targeting drug-loaded nanosystem by surface-modifying rhES using AuNPs. Our data indicate that AuNPs enhanced the concentration of rhES in tumours. Furthermore, rhES promoted transient vascular normalization in H22 xenograft models, reduced hypoxia and leaking, and improved pericyte coverage and blood perfusion, thereby increasing 5-FU delivery into the tumour and improving therapeutic efficiency. However, further research is needed to fully elucidate the mechanism of tumour vascular normalization.

## Materials and Methods

### Reagents and cells

rhES was provided by Simcere-Medgenn Bio-pharmaceutical Co., Ltd. (Shandong, China) and stored at 4 °C. 5-FU was purchased from Shanghai Xudong Haipu Pharmaceutical Co., Ltd. AuNPs were obtained from Shanghai Jie Ning Biotech Co., Ltd. The AuNPs exhibited an average diameter of 15 nm and were dissolved in distilled water and stored in a light-resistant container at 4 °C. Methoxy polyethylene glycol-Thiol (mPEG-Thiol; MW: 2000) was purchased from Shanghai zzbio Co., Ltd (Shanghai, China). The H22 cell line was purchased from Sun Yat-sen University, cultured in DMEM (Gibco, Life Technologies, USA) containing 10% foetal bovine serum (Gibco, Life Technologies, USA) and 1% penicillin/streptomycin (Hyclone), and maintained in a 37 °C incubator with 5% CO_2_.

### Preparation and characterization of the rhES-AuNPs-PEG

The rhES-AuNPs-PEG was prepared via a self-assembly process. A calculated amount of rhES was added to the dispersion of AuNPs, and the reaction system was incubated in the dark at 4 °C for 12 hours. The preparation was oscillated gently every 2 hours. Subsequently, mPEG-Thiol was introduced and incubated for another 4 h for *in vivo* treatment. SPR in the UV-visible spectrum was observed on a model V-570 Jasco dual-beam spectrophotometer. The hydrodynamic size distribution and zeta potential were evaluated by DLS using a Malvern Zetasizer Nano ZS (Malvern, UK). The morphology and surface structure were visualized using field emission transmission electron microscopy (FETEM) (JEM2100, JEOL, Japan).

### rhES-loading efficiency

The rhES-load efficiency was determined according to the previously described method^45^. The mixture was centrifuged at 13000 rpm for 20 min. Subsequently, the concentration of free rhES in the supernatant was determined using an ELISA method. Finally, the percentage loading of rhES on AuNPs was estimated using the following formula:


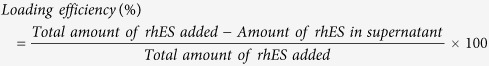


### rhES release *in vitro*

rhES release behaviour from the carrier was estimated using a centrifugal filter device (Millipore Amicon^®^ Ultra, 30-kD molecular weight cutoff). rhES-AuNPs-PEG and 10-mL PBS at different pH values were added to the filter device and centrifuge tube, respectively. The system was incubated at 37 °C for a predetermined length of time, and the filter device was centrifuged at 3000 rpm for 20 min. Next, 1 mL of PBS was withdrawn and replaced by an equal volume of fresh PBS. The rhES concentration was determined as described previously.

### Tumour xenograft models and treatment regimens

Female Kunming mice (20–40 g weight, 6–8 weeks old) were purchased from the Medical Laboratory Animal Center (Guangdong, China) and maintained under specific pathogen-free conditions. All animal study protocols were approved and conducted in accordance with the guidelines of the Laboratory Animal Ethics Committee of Jinan University.

Xenografted tumours were initiated by injection of 5 × 10^6^ H22 cells into the subcutaneous tissue of the right flank. The tumour volume was measured using a Vernier calliper every other day and calculated as V = (length × width^2^) × 0.523. Treatment with rhES-AuNPs-PEG or placebo was initiated when the tumour volume reached 175–200 mm^3^ post-inoculation and was repeated daily from day 0 to day 12. From day 1, all animals bearing tumours underwent treatment with 5-FU (40 mg/kg) daily for 4 days, followed by treatment every 2 days for 1 week. Subsequently, the mice were euthanized, and the tumours and various organs were harvested for analysis.

### *In vivo* tumour and tissue biodistribution

Mice bearing H22 tumours received rhES (10 mg/kg) or rhES-AuNPs-PEG containing the same dose of conjugated rhES for predetermined times (30 min and 4 h). The animals were sacrificed, and tissue samples from the liver, spleen, heart, lung, kidney, and tumours were collected and rapidly frozen for immunofluorescence detection using an anti-endostatin antibody (1:500; Abcam). In addition, we used optical *in vivo* imaging systems (Bruker, Germany) to detect the rhES-AuNPs-PEG biodistribution. The animals were anaesthetized with isoflurane and imaged 10 min, 30 min and 4 h after the injection of Cy5-labelled regents. The acquisition time was approximately 10 min, and image reconstruction was performed using InVivoScope 1.37 software (Bioscan).

### Vascular leakage and perfusion

Extravascular diffusion visualization was assessed by intravenous injection of 0.25 mg/mouse FITC-labelled dextran (40 -kDa; Chondrex, USA). Tumours were harvested after 20 minutes, frozen in optimal cutting temperature compound (Sakura Finetek, Torrance, CA) and stored at −80 °C. Cryosections with a thickness of 20 μm were fixed in cold acetone and rehydrated in PBS. Microvessels were stained with an anti-CD31 antibody (1:500; Abcam), and the percentage of leakage was calculated as the ratio of the dextran^+^ area to CD31^+^ area using Image J software.

Tumour blood perfusion was detected using FITC-conjugated lectin from Bandeiraea simplicifolia (Sigma-Aldrich, USA) and anti-CD31 staining. Subsequently, 10 mg/kg FITC-lectin was injected intravenously. After 1 minute, the tumours were excised, and sections were prepared as described previously. Tissue was visualized under a fluorescence microscope (Leica DM6000B), and the lectin^+^ area was presented as a percentage of the CD31^+^ area.

### Immunofluorescence

Tissues were fixed in 4% paraformaldehyde for 24 hours and paraffin-embedded, sectioned, dewaxed in xylene, and rehydrated through graded alcohols. Antigen retrieval was performed in citric acid buffer (pH 6.0). Sections were blocked in 2% normal goat serum for 1 hour and stained with primary antibodies as follows: anti-CD31 antibody (1:500; Abcam) for endothelium, α-SMA antibody (1:100; Proteintech) for pericytes, and HIF-1α antibody (1:50; Proteintech) for hypoxia. The sections were then washed and incubated with rhodamine-conjugated goat anti-rat IgG (H + L) (1:50; Proteintech) or goat anti-rabbit IgG-FITC (1:200; Santa Cruz) for 40 min at RT. The level of pericyte coverage was presented as a percent of the length along CD31^+^ vessels. The ratio of hypoxia in the tumour sections was presented as HIF-1α staining area/CD31^+^ area.

### HPLC quantification

The concentration of 5-FU in different organs was quantified using an Agilent 1100 series HPLC (Agilent^®^ Technologies, Santa Clara, CA, USA) on a COSMOSIL C18 column (250 mm × 4.5 mm, 5 μm) (Shimadzu, Tokyo, Japan). The eluents were methanol, 0.1% trifluoroacetic acid and deionized water (3:40:57, v/v/v), and the flow rate was 1.0 mL/min. 5-FU was detected at a wavelength of 265 nm. All responses obtained were analysed using Agilent^®^ ChemStation^®^ software.

### Statistical analysis

All data are presented as the mean ± standard error of the mean of independent triplicate samples. Statistics for different groups were compared by one-way analysis of variance or unpaired Student’s *t*-test. Survival time was determined with the Kaplan–Meier test. All statistical analyses were performed using GraphPad Prism (version 5.0; GraphPad Software, La Jolla, CA). *P* values less than 0.05 were considered statistically significant (*P < 0.05, **P < 0.01, and ***P < 0.001).

## Additional Information

**How to cite this article**: Li, W. *et al*. Gold Nanoparticle-Mediated Targeted Delivery of Recombinant Human Endostatin Normalizes Tumour Vasculature and Improves Cancer Therapy. *Sci. Rep.* **6**, 30619; doi: 10.1038/srep30619 (2016).

## Supplementary Material

Supplementary Information
