# Supplementary material for: Gold Nanoparticle–Mediated Targeted Delivery of Recombinant Human Endostatin Normalizes Tumour Vasculature and Improves Cancer Therapy
Source: Sci Rep. 2016 Jul 29;6:30619. doi: 10.1038/srep30619 (PMC4965746; doi:10.1038/srep30619)
Supplement: Supplementary Information [file srep30619-s1.docx]

**Gold Nanoparticle–Mediated Targeted Delivery of Recombinant Human Endostatin Normalizes Tumour Vasculature and Improves Cancer Therapy**

Wei Li^1^, Xiaoxu Zhao^1^, Bin Du^2^, Xin Li^1^, Shuhao Liu^1^, Xiao-Yan Yang ^1^, Hui Ding^1^, Wende Yang^1^, Fan Pan^1^, Xiaobo Wu^1^, Li Qin^3^, Yunlong Pan^1*^


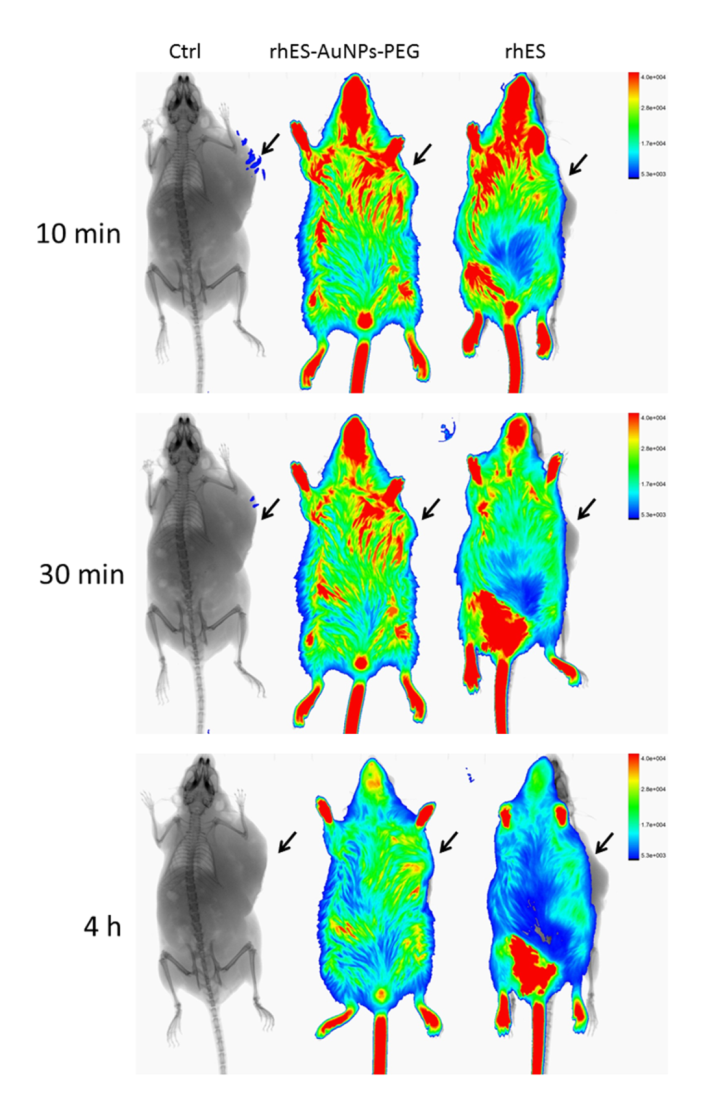


**Figure S1. In vivo tumour imaging using Cy5-labelled regents.** Cy5-conjugated rhES, rhES-AuNPs-PEG after 10min, 30 min and 4 h. As shown in the figure, Cy5-labelled rhES-AuNPs-PEG rapidly accumulated in the tumour, liver and spleen after i.v. injection within 30 min, and the levels were maintained for 4 h. By contrast, the Cy5-labeled rhES exhibited a poor specific organization distribution at 30 min and 4 h. Arrows indicate the tumour region.
